# Serious Fungal Diseases: Assessing the Impact of Training Clinicians and Laboratory Scientists in Nigeria

**DOI:** 10.1093/ofid/ofag442

**Published:** 2026-07-29

**Authors:** Rita Oladele, Damilola Akinlawon, Alexander Jordan, Iriagbonse Osaigbovo, Olufunmilola Makanjuola, Ubong A Udoh, Philip Nwajiobi-Princewill, Yahaya Mohammed, Folake Peters, Tochi Okwor, Akin Osibogun, Folasade Ogunsola, Martin Hoenigl, Jean-Pierre Gangneux, Shawn R Lockhart, Tom Chiller, Nancy A Chow

**Affiliations:** Department of Medical Microbiology and Parasitology, College of Medicine, University of Lagos, Lagos, Nigeria; Department of Community Health and Primary Care, Lagos University Teaching Hospital, Lagos, Nigeria; Mycotic Diseases Branch, Division of Foodborne, Waterborne, and Environmental Diseases, Centers for Disease Control and Prevention, Atlanta, Georgia, USA; Department of Medical Microbiology, College of Medical Sciences, University of Benin, Benin, Nigeria; Department of Medical Microbiology and Parasitology, University of Ibadan, Ibadan, Nigeria; Department of Medical Microbiology and Parasitology, University of Calabar, Calabar, Nigeria; Department of Medical Microbiology and Parasitology, National Hospital, Abuja, Nigeria; Department of Medical Microbiology and Parasitology, College of Medical Sciences, Usmanu Danfodiyo University Teaching Hospital, Sokoto, Nigeria; Department of Medical Microbiology and Parasitology, Lagos University Teaching Hospital, Lagos, Nigeria; Antimicrobial Resistance/Infection Prevention and Control Unit, Nigeria Centre for Disease Control, Abuja, Nigeria; Department of Community Health and Primary Care, College of Medicine University of Lagos, Lagos, Nigeria; Department of Medical Microbiology and Parasitology, College of Medicine, University of Lagos, Lagos, Nigeria; Division of Infectious Diseases, Department of Internal Medicine, Medical University of Graz, Graz, Austria; ECMM Excellence Center for Medical Mycology, Translational Medical Mycology Research Unit, Medical University of Graz, Graz, Austria; Centre National de Référence des Mycoses et Antifongiques LA-AspC Aspergilloses Chroniques, European Excellence Center for Medical Mycology (ECMM EC), Centre Hospitalier Universitaire de Rennes, Rennes, France; IRSET (Institut de Recherche en Santé, Environnement et Travail), UMR_S 1085, Inserm, Université de Rennes, CHU Rennes, École des Hautes Études en Santé Publique (EHESP), Rennes, France; Mycotic Diseases Branch, Division of Foodborne, Waterborne, and Environmental Diseases, Centers for Disease Control and Prevention, Atlanta, Georgia, USA; Mycotic Diseases Branch, Division of Foodborne, Waterborne, and Environmental Diseases, Centers for Disease Control and Prevention, Atlanta, Georgia, USA; Mycotic Diseases Branch, Division of Foodborne, Waterborne, and Environmental Diseases, Centers for Disease Control and Prevention, Atlanta, Georgia, USA; The members of Nigeria Fungal Diseases Surveillance and Capacity Building (NIFUSCAB) program are listed in the Supplementary Data

**Keywords:** clinicians, fungal diseases, impact, laboratory scientists, training

## Abstract

**Background:**

Surveillance for serious fungal diseases (SFDs) relies on the capacity to recognize them and request and accurately perform diagnostic investigations. However, knowledge in resource-limited settings is suboptimal. This study evaluated the impact of a training intervention on the diagnosis and management of SFDs among clinicians and laboratory scientists in Nigerian tertiary hospitals.

**Methods:**

Clinicians in 6 hospitals were purposively selected and trained in 6 SFDs using standardized modules developed by international medical mycology experts. A quasi-experimental design assessed performance using a pretest and posttest. Designated laboratory scientists received hands-on training with pretraining and posttraining assessments. Paired *t* tests were used to assess differences in mean scores. An impact assessment of the laboratory mycology records was performed 1 year after intervention using analysis of variance.

**Results:**

A total of 149 clinicians and 8 laboratory scientists participated. Mean performance scores of participants improved significantly after training, with the following percentage increases: invasive aspergillosis (25.4%), chronic pulmonary aspergillosis (13.0%), invasive candidiasis (23.8%), *Pneumocystis* pneumonia (22.6%), mucormycosis (29.5%), and histoplasmosis (20.6%) (all *P* < .001). There was also a significant improvement in the laboratory scientists’ posttraining performance (59.1%; *P* < .001). The laboratory mycology records reviewed 1 year later indicated a significant increase in the number of samples sent to the laboratory for mycological testing (*P* < .001) and the number and distribution of fungal isolates identified (*P* < .001).

**Conclusions:**

Training significantly improved SFD diagnosis and enhanced surveillance and patient care. Expanding this model across other low-resource settings is recommended.

Serious fungal diseases (SFDs) are a significant cause of disease and death in diverse patient groups, particularly the immunocompromised, and they disproportionately affect populations that are already disadvantaged by the conditions in which they are born, grow, work, live and age, the so-called social determinants of health [1, 2]. They also hinder the successful outcome of otherwise life-saving medical treatments and procedures [3]. Their clinical presentations are not specific, and their disease severity often depends on the integrity of the host's immune response; thus, a high degree of suspicion on the part of the managing clinician is needed for the early diagnosis and optimal management of these infections [3]. Our understanding of the impact of SFDs on public health in low- and middle-income countries (LMICs) in Africa and beyond remains poor due to lack of research, surveillance, diagnostic tools, and human resources in clinical and diagnostic mycology and low awareness of these diseases among healthcare personnel and the lay public [4, 5].

Nigeria has a population estimated at >216 million people and a human immunodeficiency virus (HIV) prevalence of 2.1%, with a significant proportion of those with HIV (33%) meeting the advanced HIV disease (AHD) classification [6, 7]. AHD is a well-established risk factor for a number of SFDs—namely, cryptococcal meningitis, *Pneumocystis* pneumonia, and disseminated histoplasmosis, among others [8]. In addition, Nigeria is ranked sixth among the high- tuberculosis-burden countries, and prior tuberculosis infection is a well-documented risk factor for the development of chronic pulmonary aspergillosis [9]. Tertiary-level health facilities treat patients with serious and complex underlying diseases, whose management often results in immunosuppression, a risk factor for the development of classic healthcare-related yeast and mold infections, such as candidemia and invasive aspergillosis [10].

An estimated 11.8% of Nigerians are expected to be afflicted with an SFD annually [8], but despite this projected incidence, there is paucity of data on these diseases [11, 12]. Although the true burden remains obscure, the scourge of these infections is not in doubt, as a few recent studies aimed at understanding the epidemiology of SFDs in Nigeria have provided insight on their public health impact [13]. Studies have also revealed gaps in the laboratory diagnostic capacity and poor awareness of SFDs among physicians in Nigeria [14, 15]. The Nigeria Fungal Diseases Surveillance and Capacity Building program is aimed at capacity building and conducting SFD surveillance in Nigeria. The project recognized that for the surveillance to be successful, targeted capacity building for clinicians on the diagnosis and management of SFDs was critical. Intensive “hands-on” capacity building was also done for the laboratory personnel at the selected sites for surveillance, and essential diagnostics for mycological investigations were provided. This report describes the training outcome and impact of capacity building and system strengthening.

## METHODS

This study used a quasi-experimental (1-group pretest–posttest) design to evaluate the impact of targeted training interventions on the diagnosis and management of SFDs among clinicians and laboratory scientists in Nigerian tertiary hospitals. The design was chosen to assess changes in knowledge, skills, and diagnostic performance before and after a structured training. The intervention comprised 2 interconnected components: (1) a training for clinicians focused on 6 major invasive and pulmonary fungal diseases—invasive candidiasis, invasive aspergillosis, chronic pulmonary aspergillosis, *Pneumocystis* pneumonia, mucormycosis, and histoplasmosis—delivered using standardized international modules and (2) a hands-on laboratory training for medical laboratory scientists at the National Mycology Reference Laboratory, emphasizing internationally recognized standard operating procedures for fungal diagnostics.

### Sampling Technique and Training Intervention

Purposive sampling was used to select targeted clinicians who commonly managed the at-risk population for SFDs. These clinicians were specialists selected from 6 federal tertiary hospitals located in urban areas in the country. The following specialties were involved: intensive care medicine/anesthesia, pediatrics, dermatology, otorhinolaryngology, general surgery, hematology, infectious diseases, internal medicine, medical microbiology, nephrology, radiation oncology, and family medicine. This training was reviewed and approved by the chief medical directors of the individual hospitals. The heads of departments were then requested to nominate ≥2 clinicians (preferably a senior and a junior clinician) from their department. Hospitals were also requested to nominate 2 laboratory scientists from their medical microbiology laboratory to be sent to the national mycology reference laboratory for hands-on training. The training was conducted using training modules that were developed by experts from European Confederation of Medical Mycology, Global Action for Fungal Infections, and the International Society for Human & Animal Mycology, with technical support from the CDC Mycotic Diseases Branch in Atlanta, Georgia. A “training of trainers” was then conducted for selected specialists on each of the 6 SFDs.

The specialists included an intensivist for invasive candidiasis, an otolaryngologist for mucormycosis, a pulmonologist for chronic pulmonary aspergillosis, an HIV or infectious diseases physician for *Pneumocystis* pneumonia, a hematologist or medical microbiologist for histoplasmosis, and a hematologist, oncologist, infectious diseases physician, or microbiologist for invasive aspergillosis. After the training of trainers, these trained specialists facilitated the sessions within their respective centers using the same standardized training modules, presentation slides, and assessment tools. This structured cascade model ensured uniform content delivery, reduced instructor-related bias, and maintained comparability of outcomes across all study sites.

The training consisted of 2 days of didactic lectures for the clinicians, with case series included. Structured questionnaires containing questions on the diagnosis and management of the various SFDs were administered before and after each of the respective training sessions (as a pretest and posttest). Each questionnaire had 20 questions divided into 3 sections covering overview, diagnosis, and treatment; answers were “true” or “false.”

For the laboratory scientists, an intensive 1-week “hands-on” training was conducted at the National Mycology Reference Laboratory of Nigeria. At the beginning of the training, each laboratory scientist was given fungal isolates in plates and slides to identify. Fungal serological tests were also included. This was followed by intensive, hands-on sessions conducted using internationally recognized standard operating procedures for the laboratory diagnosis of major fungal infections, including candidiasis, aspergillosis, cryptococcosis, histoplasmosis, mucormycosis, and *Pneumocystis* pneumonia. After completion of the practical sessions, a posttraining competency assessment was administered to evaluate improvement in diagnostic proficiency and procedural accuracy.

To complement the training evaluation, a baseline mycology record review was conducted to assess existing diagnostic activity and case detection trends for 2 years prior to the intervention (2020 and 2021). A follow-up record review was then performed 1 year after the training (from 16 June 2022 to 15 June 2023) to determine the impact of the capacity-building efforts on laboratory surveillance and diagnostic yield. Data were captured on the number of samples sent annually to the laboratory for mycological testing, sample types, common pathogens isolated, and serological test results.

Because the study did not involve human participants, patient consent was not required. All necessary institutional and ethical approvals were obtained prior to commencement of the study, which was reviewed and approved by the institutional review boards of all participating hospitals. The study adheres to all applicable national standards.

### Data Analysis

A correct response was awarded 1 point, and a wrong response, 0 points. The pretest and posttest performance for each question was determined by summing all correct responses for a question and presenting the sum as a percentage of the highest score attainable (which would be every participant responding correctly). Similarly, pretest and posttest performance in each section of the questionnaires was determined by summing all correct responses in the section and presenting that sum as a percentage of the highest score attainable in each section. For the laboratory scientists’ assessment, it was scored by giving 1 point for a correct identification or procedure and 0 points for an incorrect one, and their total was scored in percentages.

We computed the mean performance of participants before taking the training by dividing the total score by the number of responding participants to obtain the pretest mean scores. This computation was repeated after the training (posttest) to derive the mean posttest scores. Mean pretest scores were compared with mean posttest scores. Paired *t* tests were used to test for statistical significance in clinicians’ and laboratory scientists’ training scores. One-way analysis of variance was used to test for significant change in the mycology record review. Differences were considered significant at *P* < .05.

## RESULTS

Eight laboratory scientists from the selected tertiary facilities participated in the competency assessment, with a response rate of 100%. Of 162 clinicians, 149 voluntarily participated in the pretest and posttest (a response rate of 92%). The mean age (SD) of the clinicians was 40.4 (6.7) years, with a range of 29–59 years. The highest number of clinicians, 71 (47.7%) were in the 30–39-year age range. The majority of participants, 97 (65.1%), were men, and 86 (57.7%) had practiced medicine for 10–19 years. The specialties were anesthesia 13 (8.7%), dermatology 5 (3.4%), otorhinolaryngology 12 (8.1%), family medicine 15 (10.1%), hematology 12 (8.1%), infectious diseases 3 (2.0%), internal medicine 11 (7.4%), microbiology 32 (21.5%), nephrology 3 (2.0%), ophthalmology 2 (1.3%), pediatrics 17 (11.4%), pulmonology 11 (7.4%), radiation-oncology 7 (4.7%), and surgery 6 (4.0%) (Table 1).

**Table 1.** Sociodemographic Characteristics of Studied Clinicians

Characteristic	Clinicians, No. (%) (n = 149)
Age group, years	
20–29	1 (0.7)
30–39	71 (47.7)
40–49	63 (42.3)
50–59	14 (9.4)
Age range, years	29–59
Mean age (SD), years	40.4 (6.7)
Sex	
Male	97 (65.1)
Female	52 (34.9)
Duration of practice, years	
1–9	46 (30.9)
10–19	86 (57.7)
≥20	17 (11.4)
Range of durations, years	2–36
Mean duration (SD), years	12.4 (6.3)
Specialty	
Anesthesia	13 (8.7)
Dermatology	5 (3.4)
Otorhinolaryngology	12 (8.1)
Family medicine	15 (10.1)
Hematology	12 (8.1)
Infectious diseases	3 (2.0)
Internal medicine	11 (7.4)
Medical microbiology	32 (21.5)
Nephrology	3 (2.0)
Ophthalmology	2 (1.3)
Pediatrics	17 (11.4)
Pulmonology	11 (7.4)
Radiation oncology	7 (4.7)
Surgery	6 (4.0)

### Evaluation of the Training Courses

There was a significant improvement in the performance of the laboratory scientists after training (mean score [SD], 44.7 [20.2] before and 71.1 [6.9] after training; *P* < .001). The mean (SD) performance scores of the clinicians improved significantly with reduced variation, after trainings on all the modules (invasive aspergillosis, 12.6 [2.6] for pretest vs 15.8 [2.1] for posttest; chronic pulmonary aspergillosis, 15.4 [2.2] vs 17.4 [1.6], respectively; invasive candidiasis, 14.7 [2.1] vs 18.2 [1.1]; *Pneumocystis* pneumonia, 13.3 [2.3] vs 16.3 [1.8]; mucormycosis, 13.2 [2.2] vs 17.1 [1.7]; and histoplasmosis, 13.1 [2.4] vs 15.8 [1.8]; all *P* < .001) (Table 2). The comparative distribution of pretest and posttest scores indicated a marked improvement after training.

**Table 2.** Evaluation of Clinicians’ and Laboratory Scientists’ Training and Subsections

Training	Participants, No.	Highest Attainable Score	Mean Score (SD)		Improvement, %	*P* Value
			Pretraining	Posttraining		
Clinician training						
Invasive aspergillosis	110	20	12.6 (2.6)	15.8 (2.1)	25.4	<.001^a^
Chronic pulmonary aspergillosis	122	20	15.4 (2.2)	17.4 (1.6)	13.0	<.001^a^
Invasive candidiasis	109	20	14.7 (2.1)	18.2 (1.1)	23.8	<.001^a^
*Pneumocystis* pneumonia	114	20	13.3 (2.3)	16.3 (1.8)	22.6	<.001^a^
Mucormycosis	132	20	13.2 (2.2)	17.1 (1.7)	29.5	<.001^a^
Histoplasmosis	85	20	13.1 (2.4)	15.8 (1.8)	20.6	<.001^a^
Subsections						
Invasive aspergillosis						
Overview	110	6	5.25 (1.2)	5.57 (1.1)	6.1	.001^a^
Diagnosis	110	7	2.47 (1.5)	4.64 (1.3)	87.9	<.001^a^
Treatment	110	7	4.91 (1.3)	5.61 (1.1)	14.3	<.001^a^
Chronic pulmonary aspergillosis						<.001^a^
Overview	122	4	3.51 (0.8)	3.81 (0.6)	8.5	<.001^a^
Diagnosis	122	8	6.62 (0.9)	7.16 (0.9)	8.2	<.001^a^
Treatment	122	8	5.27 (1.5)	6.43 (1.1)	22.0	<.001^a^
Invasive candidiasis						
Overview	109	3	1.90 (0.8)	2.72 (0.6)	43.2	<.001^a^
Diagnosis	109	10	7.93 (1.2)	8.89 (0.7)	12.1	<.001^a^
Treatment	109	7	4.83 (1.3)	6.62 (0.7)	37.1	<.001^a^
*Pneumocystis* pneumonia						
Overview	114	6	3.93 (1.2)	4.94 (0.9)	25.7	<.001^a^
Diagnosis	114	6	3.22 (1.0)	3.98 (1.10)	23.6	<.001^a^
Treatment	114	8	6.14 (1.3)	7.35 (1.0)	19.7	<.001^a^
Mucormycosis						
Overview	132	5	3.17 (0.8)	4.58 (0.6)	44.5	<.001^a^
Diagnosis	132	6	3.00 (1.0)	4.27 (0.9)	42.3	<.001^a^
Treatment	132	9	7.00 (1.2)	8.20 (1.0)	17.1	<.001^a^
Histoplasmosis						
Overview	85	3	2.00 (0.9)	2.74 (0.8)	37.0	.001^a^
Diagnosis	85	9	5.42 (1.4)	6.53 (1.1)	20.5	.006^a^
Treatment	85	8	5.60 (1.5)	7.18 (0.8)	28.2	<.001^a^
Laboratory training	8	100	44.7 (20.2)	71.1 (6.9)	59.1	.002^a^

^a^Statistically significant at *P* < .05.

Further assessment revealed that in the clinician training, there was a significant increase in posttraining performance for all the assessment subsections, the overview/general, diagnosis, and treatment subsections across all the clinician training modules (for invasive aspergillosis, *P* ≤ .001 for each subsection; chronic pulmonary aspergillosis, invasive candidiasis, *Pneumocystis* pneumonia, and mucormycosis, *P* < .001 for each; and histoplasmosis, *P* ≤ .006 for each)] (Table 2). Visual charts showing details of the effect of training on the overall knowledge and knowledge of each subsection of the training modules demonstrated improved performance on a vast majority of questions and all the sections, including the overview/general, diagnosis, and treatment sections, across all the training topics (Supplementary Figures 1 and 2).

Posttraining performance improvement was also seen across all specialties. Significantly increased posttest scores were seen among family physicians (*P* ≤ .02 for each module), hematologists and radiation oncologists (*P* ≤ .003 for each module), internists (*P* ≤ .001 for each module), medical microbiologists (*P* ≤ .004 for each module), and pediatricians (*P* ≤ .02 for each module) across the 6 training modules, while anesthetists/intensivists (*P* ≤ .02 each) and surgeons (*P* ≤ .003 each) had significantly improved posttest scores on the invasive aspergillosis, chronic pulmonary aspergillosis, invasive candidiasis, *Pneumocystis* pneumonia, and mucormycosis modules (Table 3). Performance stratification across sites of training also revealed improved posttraining performance, which was largely significant across all modules in all sites (*P* < .01), other than the invasive aspergillosis training in site 2 and the chronic pulmonary aspergillosis training in site 5, which both had notably but not significantly increased posttraining performance scores (Supplementary Table 1).

**Table 3.** Comparison of Pretest and Posttest Performance of Various Specialties Involved in Clinicians’ Training

Specialty^a^	Mean Score (SD)					
	Invasive Aspergillosis	Chronic Pulmonary Aspergillosis	Invasive Candidiasis	*Pneumocystis* Pneumonia	Mucor mycosis	Histoplasmosis
Anesthesia/intensive care	n = 10	n = 10	n = 10	n = 10	n = 11	n = 8
Pretest	12.8 (2.7)	14.4 (2.1)	14.5 (2.7)	12.7 (1.8)	11.7 (2.4)	12.5 (3.6)
Posttest	16.8 (2.3)	17.1 (2.1)	17.3 (1.5)	16.3 (2.4)	16.9 (1.8)	15.5 (2.7)
*P* value	.006^b^	.01^b^	.02^b^	<.001^b^	<.001^b^	.08
Family medicine	n = 14	n = 13	n = 11	n = 14	n = 15	n = 10
Pretest	12.4 (2.6)	15.1 (2.0)	14.3 (1.8)	12.6 (3.1)	12.7 (2.4)	12.3 (3.2)
Posttest	15.1 (2.7)	16.9 (1.7)	18.5 (1.1)	16.0 (2.2)	16.3 (2.3)	15.6 (2.1)
*P* value	.002^b^	.001^b^	<.001^b^	<.001^b^	<.001^b^	.02^b^
Hematology/radiation oncology	n = 13	n = 17	n = 11	n = 13	n = 18	n = 11
Pretest	12.3 (3.4)	15.3 (1.7)	14.5 (1.9)	12.2 (2.5)	13.6 (1.7)	12.2 (1.5)
Posttest	15.7 (1.6)	17.3 (1.6)	18.3 (0.9)	15.9 (1.4)	16.7 (1.6)	15.9 (1.9)
*P* value	.003^b^	.00^b^	<.001^b^	<.001^b^	<.001^b^	<.001^b^
Internal medicine^c^	n = 22	n = 9	n = 21	n = 27	n = 31	n = 19
Pretest	13.4 (1.5)	16.0 (2.2)	15.1 (2.3)	14.4 (1.6)	13.3 (2.3)	14.1 (2.0)
Posttest	16.0 (2.1)	17.8 (1.7)	18.1 (1.4)	16.7 (1.7)	17.2 (1.2)	16.3 (1.5)
*P* value	<.001^b^	<.001^b^	<.001^b^	<.001^b^	<.001^b^	<.001^b^
Medical microbiology	n = 23	n = 25	n = 26	n = 29	n = 26	n = 20
Pretest	13.2 (2.7)	15.7 (2.5)	15.2 (2.1)	13.7 (2.2)	13.9 (2.1)	13.2 (1.8)
Posttest	15.4 (2.3)	17.4 (1.7)	18.3 (0.9)	16.9 (1.4)	17.3 (1.3)	15.8 (1.7)
*P* value	<.001^b^	.004^b^	<.001^b^	<.001^b^	<.001^b^	<.001^b^
Pediatrics	n = 11	n = 14	n = 10	n = 9	n = 14	n = 8
Pretest	10.9 (2.7)	15.5 (2.2)	14.6 (1.6)	13.7 (1.5)	12.5 (1.7)	13.4 (2.1)
Posttest	16.8 (1.3)	17.4 (1.7)	19.0 (0.8)	15.1 (0.8)	16.6 (1.9)	15.8 (1.2)
*P* value	<.001^b^	.03^b^	<.001^b^	.02^b^	<.001^b^	.01^b^
Surgery^d^	n = 17	n = 18	n = 15	n = 12	n = 17	n = 9
Pretest	12.3 (2.6)	14.9 (2.6)	13.7 (1.9	11.9 (2.4)	13.5 (2.5)	13.2 (3.0)
Posttest	15.5 (1.8)	17.6 (1.4)	18.2 (0.9)	16.0 (3.4)	17.8 (2.1)	15.1 (1.5)
*P* value	<.001^b^	<.001^b^	<.001^b^	.003^b^	<.001^b^	.10

^a^The number of observations/clinicians involved.

^b^Statistically significant at *P* < .05.

^c^Internal medicine includes pulmonology, infectious diseases, nephrology, and dermatology.

^d^Surgery includes otorhinolaryngology, general surgery, and ophthalmology.

### Impact of Capacity Building and Systems Strengthening

The number of samples sent to the laboratory for both culture and serological testing rose dramatically. In the 1-year period after intervention, 1281 samples were received for mycological testing, which was significantly higher than annual preintervention numbers for both 2020 (400 samples) and 2021 (529 samples) (*P* < .001). The number of positive results also increased significantly (*P* < .001) in the 1 year after intervention; there were 504 positive results from both culture and serology, compared with the preintervention years, 2020 (151 positives) and 2021 (257 positives). The proportion of positives per sample sent remained largely unchanged over the years, indicating that diagnosable fungal infections had likely been missed before the training.

Regarding fungal cultures, there was a significant increase in the number of fungal isolates identified (*P* < .001). A total of 179 *Candida* spp (*P* < .001) and 12 *Aspergillus* spp (*P* = .01) isolates were isolated from invasive site samples (blood, urine, vitreous tap, biopsy specimens, etc) in the 1 year after intervention, significantly surpassing preintervention figures. The positivity rates for cultures were also higher for *Candida* spp (22.9% [179 of 783]) and *Aspergillus* spp (1.5% [12 of 783]), compared with the rates in the preintervention years for both *Candida* spp (5.9% [23 of 388] for 2020 and 4.8% [25 of 526] for 2021; *P* < .001) and *Aspergillus* spp (0% [0 of 388)] and 0.4% [2 of 526], respectively; *P* = .01). Furthermore, there was a marked increase in *Candida* bloodstream infections diagnosed during the 1-year postintervention period, with 48 of 76 cases of candidemia (63.2%) identified in the laboratories, (*P* < .001) (Table 4).

**Table 4.** Impact of Clinician and Laboratory Scientist Training on Mycology Record Review in Nigerian Tertiary Laboratories Involved in Capacity Building

Result	Preintervention		Postintervention: 2022–2023	*P* Value
	2020	2021		
Total mycology samples, no.	400	529	1281	<.001^a^
Positive mycology results, no. (%)	152 (38.0)	257 (48.6)	616 (48.1)	<.001^a^
Cultures				
Total no.	388	526	783	.001^a^
Positive cultures, no. (%)	151 (38.9)	257 (48.9)	504 (64.4)	<.001^a^
*Candida* spp isolated from invasive samples, no. (%)^b^	23 (5.9)	25 (4.8)	179 (22.9)	<.001^a^
*Aspergillus* spp isolated from invasive samples, no. (%)^c^	0 (0)	2 (0.4)	12 (1.5)	.01^a^
Blood cultures, no.	6	25	76	<.001^a^
Blood cultures positive for candidemia, no. (%)	1 (16.7)	21 (84.0)	48 (63.2)	<.001^a^
Serological results, no.				
*Aspergillus* IgG positive	0	0	42	<.001^a^
Galactomannan positive	0	0	55	<.001^a^
CrAg positive^d^	1	0	13	<.001^a^

Abbreviations: CrAg, cryptococcal antigen; IgG, immunoglobulin G.

^a^Statistically significant at *P* < .05.

^b^Invasive samples for *Candida* spp included blood, urine, vitreous tap, and biopsy specimens.

^c^Invasive samples for *Aspergillus* spp included biopsy and vitreous tap specimens.

^d^CrAg results here are from individuals without human immunodeficiency virus (HIV), thus excluding those domiciled with the national program for advanced HIV disease.

Before the capacity-building activities, no fungal serological investigations were being performed in any of the selected facilities. Following the intervention, significant increases (*P* < .001 each) were observed, with 42 *Aspergillus* immunoglobulin G and 55 *Aspergillus* galactomannan positive results. In addition, cerebrospinal fluid cryptococcal antigen (CrAg) positives rose significantly (*P* < .001), with 13 positive samples recorded in the postintervention year compared with 1 in 2020 and 0 in 2021 (Table 4).

## DISCUSSION

Understanding the burden of fungal diseases and their impact on public health requires disease surveillance. Surveillance, in turn, relies on disease recognition, which requires not only laboratory diagnostic capacity but also that clinicians have a high index of suspicion to consider fungal infections and request the appropriate specimen collection and diagnostic testing. Educational programs focused on mycoses have been demonstrated to bridge knowledge gaps among physicians and other health workers in developing countries [16, 17]. This report presents findings from a capacity-building program designed to improve the diagnosis of SFDs in Nigeria.

The clinicians involved in the training at the sites were not randomly selected; instead, they were targeted specialists who manage populations at high risk for SFDs. Family physicians were also included as they serve as the first point of contact for these patients. Their role is critical in relying on a high index of suspicion to request necessary diagnostics, refer patients to appropriate specialists, and initiate timely management. This approach was essential for improving prognosis, as many of these conditions are time sensitive and require early diagnosis for appropriate management.

While the outcome of the training was an improved knowledge of the diagnosis and management of SFDs among the clinicians and laboratory scientists, as evidenced by their significantly improved posttraining test performance, the impact of this training also translated into an increase in the frequency of SFDs diagnosed (in symptomatic patients), revealed by the significant increase in recorded figures of both cultures and serological testing data. Our findings revealed that the number of mycology samples sent for different fungal studies and the numbers of isolates/positive samples discovered were significantly increased (both *P* < .001). Serological data were initially nonexistent prior to the intervention, but after the intervention they have demonstrated significant and impactful results, highlighting the enhanced diagnostic capacity achieved. Serological investigations captured in this study, such as *Aspergillus* galactomannan and immunoglobulin G, are point-of-care tests with rapid result times and satisfactory levels of reliability in detecting SFDs. These parameters would most likely have contributed substantially to the enhancement of patient outcomes due to the enhanced capacity for disease detection, noting that the training received encompassed both their identification and their management.

A study performed in 1 tertiary facility in Nigeria over the space of 1 year, sampling a total of 230 patients who had pyrexia, revealed that 5.21% had candidemia [18]. This study showed a high rate of positive sampling, as 48 patients (63.2%) were seen to have candidemia, of 76 suspected cases with blood cultures obtained. This reveals the effect of focused training of clinicians on these conditions in order to increase their capacity to make accurate diagnosis. Despite this report, the true burden of these entities remains largely unquantified [19, 20]. This report is a clear indication that, proportionately as more testing is performed, more fungi are diagnosed; the increase in testing did not decrease the proportion of positive tests.

While a 2023 review of mucormycosis in Africa spanning 6 decades revealed only 6 cases documented in the literature, an obvious underdiagnosis, epidemiological data on invasive aspergillosis and *Pneumocystis* pneumonia are virtually nonexistent [21]. This lack of high-quality data obfuscates the public health relevance of SFDs, which are associated with high morbidity and mortality rates and economic costs, underscoring the need for interventions like this, involving capacity building and routine surveillance to better track cases and understand their impact on the Nigerian populace and that of other LMICs [13].

The provision of fungal diagnostics (for both culture and serology) has substantially improved the availability of mycology data in hospitals, providing critical insights into the burden of serious fungal infections. To sustain these gains, the consistent supply of essential diagnostics, coupled with ongoing training for healthcare personnel, should be prioritized to enhance the positive outcomes of such interventions. Such long-term impact has been previously shown in Nigeria, where similar training on cryptococcal meningitis and system strengthening brought about improved diagnosis among patients with AHD [16]. In Colombia also, a significant increase in the diagnosis of histoplasmosis was demonstrated after an educational initiative was executed in the country [17]. These notable examples each had a single disease focus, while our index program targeted multiple SFDs. It is noteworthy that the CrAg tests highlighted in the current study involved individuals without HIV, distinguishing them from the country's AHD platform, where CrAg testing is typically conducted for individuals living with HIV.

Knowledge should never be assumed, and clinicians should undergo training and retraining. For instance, in this study, the training on *Pneumocystis* pneumonia led to significantly improved performance after training, despite the disease being an HIV-defining condition and a regular focus for many of these clinicians who manage people living with HIV. Laboratory diagnostic support should be broadened urgently to encompass diseases like *Pneumocystis* pneumonia, as most LMICs currently lack the necessary capacity to diagnose this condition. Furthermore, the implementation of a national strategy for an external quality assessment program for laboratories maintains skills.

The limitation of this study was in its quasi-experimental design, as since there was no control group, we were unable to compare how the direction and extent of observed changes from pretest to posttest differed from changes in a group that did not receive the training. Moreover, different sets of trainers were involved across the sites for the clinicians, which may have introduced variations in the outcome of the training on clinicians across the different sites. However, the intervention was delivered using standardized training content developed by experts in the field. This ensured that the information made available to participants across sites was uniform despite being delivered by different trainers.

The training for clinicians was conducted outside their facility to ensure that there were no distractions from their routine hospital schedules. While this approach is greatly advantageous and necessary to ensure the reduction of distractions, it may have the drawback of possible tardiness by clinicians who have demanding schedules, particularly when they are located in regions in the country laden with traffic encumbrances. This was the particular reason for varying number of attendees at the trainings and contributed to the varying number of participants in the clinicians’ assessments.

The sustainability of the training outcomes was an important consideration in the design and follow-up of this intervention. To ensure continued performance and reinforcement of acquired competencies, the project team initiated several measures to institutionalize the training framework. First, an online webinar series on SFDs has been developed and is hosted on the digital learning platform of the National Postgraduate Medical College of Nigeria, the national body responsible for postgraduate specialist training and continuous professional development. This allows ongoing access to standardized training content for new and existing resident physicians across the country, who are awarded CME points. Second, there are plans to hold an annual training course at the mycology reference laboratory for laboratory scientists. These steps are designed to ensure continued capacity building and retention of diagnostic competence across Nigeria's tertiary health system.

In conclusion, the training sessions produced significant improvements in clinicians’ and laboratory scientists’ knowledge of the diagnosis and management of SFDs, which translated to the positive impact of an increased realization of the burden of these diseases in the hospitals. Similar efforts should be adopted as a component of any approach to tackling the menace of these deadly diseases. To this end, the validated training materials could be leveraged in postgraduate medical curricula for strategic training and retraining of targeted specialists involved in the management of these diseases. Training clinicians is one thing, but having the tests available for them to order is another. Building capacity needs both components to be successful. We recommend that this capacity building and strengthening program be replicated in other LMICs.

## Supplementary Material

ofag442_Supplementary_Data

## Supplementary Data

Supplementary materials are available at *Open Forum Infectious Diseases* online. Consisting of data provided by the authors to benefit the reader, the posted materials are not copyedited and are the sole responsibility of the authors, so questions or comments should be addressed to the corresponding author.
