# Supplementary material for: Serious Fungal Diseases: Assessing the Impact of Training Clinicians and Laboratory Scientists in Nigeria
Source: Open Forum Infect Dis. 2026 Jul 29;13(8):ofag442. doi: 10.1093/ofid/ofag442 (PMC13439316; doi:10.1093/ofid/ofag442)
Supplement: Supplementary file 1 [file ofag442_supplementary_data.zip › Supp Material (Tables, Figs, Clinician survey).docx]

**SUPPLEMENTARY TABLES AND FIGURES**

Invasive aspergillosis (n = 110)

Percentage

|  | Pre-test | Post-test |
| --- | --- | --- |
| Q1 | 74.5 | 88.2 |
| Q2 | 93.6 | 94.5 |
| Q3 | 86.4 | 91.8 |
| Q4 | 83.6 | 90.9 |
| Q5 | 98.2 | 97.3 |
| Q6 | 88.2 | 94.5 |
| Q7 | 45.5 | 90.9 |
| Q8 | 28.2 | 71.8 |
| Q9 | 78.2 | 95.5 |
| Q10 | 12.7 | 45.5 |
| Q11 | 22.7 | 32.7 |
| Q12 | 50.9 | 88.2 |
| Q13 | 9.1 | 39.1 |
| Q14 | 88.2 | 90.9 |
| Q15 | 71.8 | 99.1 |
| Q16 | 40.9 | 75.5 |
| Q17 | 53.6 | 61.8 |
| Q18 | 76.4 | 84.5 |
| Q19 | 75.5 | 63.6 |
| Q20 | 84.5 | 85.5 |

Chronic Pulmonary Aspergillosis (n = 122)

Percentage

|  | Pre-test | Post-test |
| --- | --- | --- |
| Q1 | 91.8 | 95.9 |
| Q2 | 83.6 | 96.7 |
| Q3 | 95.1 | 96.7 |
| Q4 | 80.3 | 91.8 |
| Q5 | 95.1 | 100 |
| Q6 | 97.5 | 100 |
| Q7 | 98.4 | 100 |
| Q8 | 73.8 | 95.9 |
| Q9 | 86.1 | 78.7 |
| Q10 | 95.1 | 96.7 |
| Q11 | 86.9 | 84.4 |
| Q12 | 29.5 | 60.7 |
| Q13 | 46.7 | 93.4 |
| Q14 | 70.5 | 81.1 |
| Q15 | 64.8 | 78.7 |
| Q16 | 61.5 | 66.4 |
| Q17 | 44.3 | 68 |
| Q18 | 66.4 | 79.5 |
| Q19 | 82 | 77.9 |
| Q20 | 91.8 | 98.4 |

Invasive candidiasis (n = 109)

Percentage

|  | Pre-test | Post-test |
| --- | --- | --- |
| Q1 | 80.7 | 94.5 |
| Q2 | 77.1 | 92.7 |
| Q3 | 32.1 | 85.3 |
| Q4 | 99.1 | 100 |
| Q5 | 81.7 | 100 |
| Q6 | 94.5 | 99.1 |
| Q7 | 11.9 | 14.7 |
| Q8 | 99.1 | 100 |
| Q9 | 88.1 | 94.5 |
| Q10 | 62.4 | 97.2 |
| Q11 | 92.7 | 99.1 |
| Q12 | 85.3 | 96.3 |
| Q13 | 78 | 88.1 |
| Q14 | 50.5 | 88.1 |
| Q15 | 83.5 | 95.4 |
| Q16 | 96.3 | 99.1 |
| Q17 | 82.6 | 96.3 |
| Q18 | 40.4 | 93.6 |
| Q19 | 41.3 | 92.7 |
| Q20 | 88.1 | 97.2 |

Pneumocystis pneumonia (n = 114)

Percentage

|  | Pre-test | Post-test |
| --- | --- | --- |
| Q1 | 97.4 | 99.1 |
| Q2 | 38.6 | 37.7 |
| Q3 | 50.9 | 68.4 |
| Q4 | 78.1 | 93 |
| Q5 | 83.3 | 98.2 |
| Q6 | 44.7 | 97.4 |
| Q7 | 92.1 | 93.9 |
| Q8 | 21.1 | 22.8 |
| Q9 | 35.1 | 46.5 |
| Q10 | 63.2 | 86.8 |
| Q11 | 32.5 | 69.3 |
| Q12 | 78.1 | 78.9 |
| Q13 | 97.4 | 100 |
| Q14 | 86 | 93 |
| Q15 | 83.3 | 97.4 |
| Q16 | 90.4 | 88.6 |
| Q17 | 75.4 | 95.6 |
| Q18 | 43.9 | 74.6 |
| Q19 | 81.6 | 98.2 |
| Q20 | 56.1 | 87.7 |

Mucormycosis (n = 132)

Percentage

|  | Pre-test | Post-test |
| --- | --- | --- |
| Q1 | 11.4 | 81.1 |
| Q2 | 94.7 | 100 |
| Q3 | 90.2 | 98.5 |
| Q4 | 83.3 | 96.2 |
| Q5 | 37.9 | 81.8 |
| Q6 | 50 | 88.6 |
| Q7 | 88.6 | 98.5 |
| Q8 | 12.1 | 77.3 |
| Q9 | 51.5 | 28 |
| Q10 | 15.9 | 38.6 |
| Q11 | 81.8 | 96.2 |
| Q12 | 96.2 | 98.5 |
| Q13 | 30.3 | 88.6 |
| Q14 | 84.1 | 88.6 |
| Q15 | 43.9 | 87.9 |
| Q16 | 87.1 | 98.5 |
| Q17 | 97 | 99.2 |
| Q18 | 90.2 | 90.9 |
| Q19 | 77.3 | 70.5 |
| Q20 | 93.9 | 97 |

Histoplasmosis (n = 85)

Percentage

|  | Pre-test | Post-test |
| --- | --- | --- |
| Q1 | 55.3 | 65.9 |
| Q2 | 90.6 | 97.6 |
| Q3 | 70.6 | 82.4 |
| Q4 | 85.9 | 97.6 |
| Q5 | 91.8 | 95.3 |
| Q6 | 82.4 | 91.8 |
| Q7 | 87.1 | 96.5 |
| Q8 | 47.1 | 70.6 |
| Q9 | 18.8 | 28.2 |
| Q10 | 30.6 | 22.4 |
| Q11 | 70.6 | 96.5 |
| Q12 | 20 | 17.6 |
| Q13 | 35.3 | 78.8 |
| Q14 | 89.4 | 97.6 |
| Q15 | 82.4 | 98.8 |
| Q16 | 54.1 | 81.2 |
| Q17 | 55.3 | 94.1 |
| Q18 | 78.8 | 78.8 |
| Q19 | 89.4 | 96.5 |
| Q20 | 75.3 | 91.8 |

**Figure 1(Supplementary): Pretest and posttest performance of clinicians**

*Alt text: Bar chart comparing pretest and posttest scores of clinicians across six fungal disease modules: invasive aspergillosis, chronic pulmonary aspergillosis, invasive candidiasis, Pneumocystis pneumonia, mucormycosis, and histoplasmosis. All posttest scores are higher, indicating improved knowledge after training.*

%

| Invasive aspergillosis | Pretest | Posttest |
| --- | --- | --- |
| Overview/General (n = 660) | 87.4 | 92.9 |
| Diagnosis (n = 770) | 35.3 | 66.2 |
| Treatment (n = 770) | 70.1 | 80.1 |

Chronic pulmonary aspergillosis

%

|  | Pretest | Posttest |
| --- | --- | --- |
| Overview/General (n = 488) | 87.7 | 95.2 |
| Diagnosis (n = 976) | 82.8 | 89.5 |
| Treatment (n = 976) | 66 | 80.4 |

Invasive candidiasis

%

|  | Pre-test | Post-test |
| --- | --- | --- |
| Overview/ General (n = 327) | 63.3 | 90.8 |
| Diagnosis (n = 1,090) | 79.3 | 88.9 |
| Treatment (n = 763) | 68.9 | 94.6 |

Pneumocystis pneumonia

%

|  | Pretest | Posttest |
| --- | --- | --- |
| Overview/General (n = 684) | 65.5 | 82.3 |
| Diagnosis (n = 684) | 53.7 | 66.4 |
| Treatment (n = 912) | 76.8 | 91.9 |

Mucormycosis

%

|  | Pretest | Posttest |
| --- | --- | --- |
| Overview/General (n = 660) | 63.5 | 91.5 |
| Diagnosis (n = 792) | 50 | 71.2 |
| Treatment (n = 1,188) | 77.8 | 91.1 |

Histoplasmosis

%

|  | Pretest | Posttest |
| --- | --- | --- |
| Overview/General (n = 255) | 72.2 | 82 |
| Diagnosis (n = 765) | 59.3 | 68.5 |
| Treatment (n = 680) | 70 | 89.7 |

**Figure 2(Supplementary): Pretest and posttest performance sections in clinicians’ training.**

*Alt text: Grouped bar chart showing mean scores in three sections (overview, diagnosis, treatment) of clinician assessments before and after training on each of the six fungal diseases. Substantial improvements are seen across all sections and diseases post-training.*

**Supplementary Table 1: Pretest and Posttest performance of various sites**

|  |  |  | **Mean ± SD** |  |  |  |
| --- | --- | --- | --- | --- | --- | --- |
| **IFD** | **Site 1** | **Site 2** | **Site 3** | **Site 4** | **Site 5** | **Site 6** |
|  |  |  |  |  |  |  |
| **Invasive** |  |  |  |  |  |  |
| **Aspergillosis** | **n = 22** | **n = 7** | **n = 15** | **n = 19** | **n = 20** | **n = 27** |
| Pretest | 13.5 ± 1.5 | 13.9 ± 1.3 | 10.5 ± 2.9 | 12.6 ± 2.8 | 13.1 ± 2.8 | 12.4 ± 2.6 |
| Posttest | 16.1 ± 2.2 | 16.1 ± 2.1 | 15.0 ± 2.5 | 15.8 ± 2.1 | 16.1 ± 1.9 | 15.7 ± 1.9 |
| p value | **0.000*** | **0.094** | **0.000*** | **0.000*** | **0.000*** | **0.000*** |
|  |  |  |  |  |  |  |
| **Chronic pulmonary aspergillosis** | **n = 23** | **n = 12** | **n = 19** | **n = 20** | **n = 21** | **n = 27** |
| Pretest | 15.4 ± 1.9 | 16.1 ± 1.9 | 15.4 ± 2.5 | 14.4 ± 2.8 | 16.0 ± 2.1 | 15.5 ± 2.0 |
| Posttest | 17.0 ± 1.7 | 18.3 ± 1.2 | 17.7 ± 1.7 | 16.8 ± 1.7 | 17.2 ± 1.4 | 17.9 ± 1.7 |
| p value | **0.001*** | **0.004*** | **0.000*** | **0.000*** | **0.051** | **0.000*** |
|  |  |  |  |  |  |  |
| **Invasive Candidiasis** | **n = 18** | **n = 12** | **n = 18** | **n = 18** | **n = 17** | **n = 26** |
| Pretest | 13.9 ± 2.2 | 14.6 ± 1.8 | 15.2 ± 2.0 | 14.8 ± 2.0 | 15.6 ± 1.9 | 14.0 ± 2.1 |
| Posttest | 17.5 ± 1.3 | 18.2 ± 1.3 | 18.4 ± 1.1 | 18.2 ± 0.9 | 18.6 ± 1.0 | 18.4 ± 0.9 |
| p value | **0.000*** | **0.000*** | **0.000*** | **0.000*** | **0.000*** | **0.000*** |
|  |  |  |  |  |  |  |
| ***Pneumocystis* pneumonia** | **n = 21** | **n = 10** | **n = 17** | **n = 19** | **n = 20** | **n = 27** |
| Pretest | 12.7 ± 2.2 | 14.0 ± 1.7 | 12.9 ± 2.0 | 13.2 ± 2.9 | 12.8 ± 2.3 | 14.2 ± 2.1 |
| Posttest | 15.6 ± 2.3 | 17.4 ± 1.2 | 15.9 ± 1.8 | 15.5 ± 1.3 | 16.8 ± 1.5 | 16.8 ± 1.5 |
| p value | **0.000*** | **0.000*** | **0.000*** | **0.000*** | **0.001*** | **0.000*** |
|  |  |  |  |  |  |  |
| **Mucormycosis** | **n = 24** | **n = 19** | **n = 20** | **n = 22** | **n = 21** | **n = 26** |
| Pretest | 13.1 ± 2.0 | 12.4 ± 2.0 | 12.9 ± 2.5 | 13.9 ± 1.9 | 12.8 ± 2.6 | 13.1 ± 2.0 |
| Posttest | 17.4 ± 1.6 | 16.4 ± 1.7 | 16.6 ± 1.8 | 17.2 ± 2.0 | 16.8 ± 1.6 | 17.4 ± 1.6 |
| p value | **0.000*** | **0.000*** | **0.000*** | **0.000*** | **0.000*** | **0.000*** |
|  |  |  |  |  |  |  |
| **Histoplasmosis** | **n = 19** |  |  | **n = 21** | **n = 17** | **n = 28** |
| Pretest | 13.2 ± 2.7 |  |  | 13.8 ± 2.3 | 13.0 ± 2.6 | 12.6 ± 2.2 |
| Posttest | 16.5 ± 2.0 |  |  | 16.0 ± 1.5 | 16.3 ± 1.5 | 14.9 ± 1.6 |
| p value | **0.000*** |  |  | **0.001*** | **0.000*** | **0.000*** |

IFD – invasive fungal disease, *Statistically significant, n – number of observations/clinicians

*Alt text: Supplementary table presenting site-specific pretest and posttest scores across six fungal disease modules, including sample size, mean scores, and p-values for each site.*

**CLINICIAN TRAINING PRETEST/POSTEST**

**Section A: Personal information**

1. Hospital (Institution):
2. Age as at last birthday (in years)………….
3. Sex: Male [ ]	Female [ ]
4. Number of years in Practice…………….
5. Specialty …………………………..
6. Give yourself an identification e.g. initials or a number known to you ONLY ……………. This identity shall be used by you for the post-training assessment also

**Section B : Questions**

**Please answer the questions in the “Diagnosis” and “Treatment” sections below using True (T) or False (F)**

**Invasive Aspergillosis**

General

1. Invasive aspergillosis is an opportunistic infection only seen in neutropenic patients.
2. Without treatment mortality from invasive aspergillosis is 80-90%
3. Only neutropenic patients are at risk
4. Invasive aspergillosis is an angio-invasive disease
5. Early diagnosis and timely therapy significantly impact outcome
6. ICU admission is a risk factor for invasive aspergillosis

Diagnosis

7. Galactomannan test is the gold standard test
8. Culture is the gold standard test
9. There is a point of care test available with results in less than 4 hours
10. Blood culture plays a role in diagnosis
11. Urine samples for fungal studies is recommended
12. 1,3-β-D glucan test is gold standard
13. Aspergillus PCR is a confirmatory test

Treatment

14. Prophylaxis is essential in the at-risk population
15. Voriconazole is the drug of choice
16. Itraconazole is the drug of choice
17. Liposomal Amphotericin B is only for salvage therapy
18. Posaconazole can be used for targeted therapy
19. Inhalational antifungals can be used for treatment
20. Drug-drug interaction is not important when treating invasive aspergillosis

**Chronic Pulmonary Aspergillosis (CPA)**

General

1. *Aspergillus spp.* are everywhere in the environment
2. CPA is a spectrum of diseases including simple aspergilloma
3. CPA is easily misdiagnosed as tuberculosis
4. CPA is a common post tuberculosis sequalae

Diagnosis.

Clinical presentation includes;

5. Cough of more than three months duration
6. Hemoptysis
7. Dyspnoea

Diagnostic tests include;

8. Serum for *Aspergillus* IgG level
9. Aspergillus galactomannan test
10. Sputum or Bronchoalveolar lavage culture
11. Histology, biopsy or CT scan
12. Total *Aspergillus* IgE levels

Treatment

13. Itraconazole is a first line drug
14. Voriconazole is the drug of choice
15. Amphotericin is only used for salvage therapy
16. Echinocandins are not used in this condition
17. The duration of treatment is six weeks
18. If azoles are used for treatment then liver function tests must be done eight weekly
19. Chest X-ray or CT scan could be used to monitor response to therapy
20. Therapeutic drug monitoring levels are essential in treating CPA

**Invasive Candidiasis**

General

1. *Candida spp.* are the most frequently occurring invasive fungal infections.
2. *Candida spp.* are endogenous opportunistic pathogens.
3. *Candida spp.* are normal flora of the whole GIT.

Diagnosis

4. Early diagnosis of invasive candidiasis significantly impacts outcome.
5. Fever could be the only clinical manifestation.
6. History of gut surgery, ICU admission, and central line are ALL risk factors for invasive candidiasis.
7. Persons at risk include those living with tuberculosis.
8. Persons at risk include those living with diabetes.
9. The specimen for laboratory diagnosis of Candidiasis depends on the site of infection.
10. Β-D-glucan is a marker for invasive candidiasis.
11. The meninges, bladder and blood vessel are all sites where invasive candidiasis could occur.
12. Representative samples for laboratory diagnosis could include culture of urine, blood or CSF.
13. End-organ dissemination is uncommon in this condition.

Treatment

14. The most widely available and effective treatment for invasive candidiasis is ketoconazole.
15. Azoles and Echinocandins are antifungal classes both effective in invasive candidiasis treatment.
16. *Candida spp.* can express multi-drug resistance.
17. Prophylaxis of high-risk patients against invasive candidiasis is beneficial.
18. Empirical treatment when indicated is encouraged with an echinocandin as the first line drug.
19. Echinocandins are the first line recommended drug for both neutropenic and non-neutropenic patients.
20. Treatment should continue for at least two weeks after the first negative sample on investigation.

***Pneumocystis* pneumonia (PcP)**

General

1. PcP is a disease seen in children only
2. PcP is endemic in Nigeria
3. It presents commonly as productive cough
4. Oxygen saturation levels are critical in making diagnosis
5. Only seen in HIV-infected patients
6. The organism can be grown in the laboratory

Diagnosis

7. Representative samples to send to lab include bronchoalveolar lavage, sputum, nasopharyngeal aspirate, or gastric washout
8. Ground glass appearance seen on Chest CT scan is diagnostic
9. The test to request for to make a diagnosis is fungal studies
10. The test to request for to make a diagnosis is direct microscopy – GIEMSA staining
11. The B-D glucan test is diagnostic
12. Polymerase Chain Reaction (PCR) test is the gold standard

Treatment

13. Prophylaxis is essential in the at-risk population
14. The prophylaxis medication of choice is an oral cephalosporin
15. Medication for treatment is trimethoprim sulfamethoxazole
16. Treatment regimen should be given for two weeks only
17. Corticosteroids could aid therapy in some cases
18. The isolation of an infected person is critical to prevent human- human transmission
19. Pentamidine, Atovaquone and Clindamycin are good treatment alternatives
20. Voriconazole and Isavuconazole are good treatment alternatives

**Mucormycosis**

General

1. Mucormycosis is a slow, indolent disease
2. Mucormycosis is commonly associated with uncontrolled diabetes mellitus.
3. The commonest clinical presentation of mucormycosis is rhinofacial disease.
4. Cutaneous mucormycosis can affect immunocompetent people
5. The African region has the highest case reporting rate for mucormycosis worldwide

Diagnosis

6. Tissue specimens preserved in formalin produce a good yield of the agents of mucormycosis when cultured in the microbiology laboratory
7. Tissue specimens for diagnosis of mucormycosis should contain viable tissue
8. Agents of mucormycosis are slow growers and culture requires at least two weeks for growth to be observed
9. The diagnosis of mucormycosis can be made the same day specimens are sent to the laboratory
10. Histopathology is very useful for speciating the fungal agent causing mucormycosis
11. Radiology is helpful in the diagnosis of pulmonary mucormycosis

Treatment

12. Mucormycosis is best managed by endocrinologists alone
13. Voriconazole is the drug of first choice for the treatment of mucormycosis
14. Liposomal Amphotericin B is less toxic than the conventional Amphotericin B deoxycholate
15. Fluconazole is the best option for treating mucormycosis in patients with renal compromise
16. Surgery is irrelevant in the current management of mucormycosis
17. Treating the underlying condition is useful in mucormycosis management
18. Treatment for mucormycosis should not exceed two weeks
19. Mucormycosis can be treated with oral antifungal medication
20. Monitoring of renal function is mandatory when managing mucormycosis

**Histoplasmosis**

General

1. Histoplasmosis is an endemic disease in Nigeria
2. Histoplasmosis is a neglected disease
3. Nigeria antiretroviral therapy (ART) guidelines have histoplasmosis diagnosis included in the advanced HIV disease (AHD) package of care

Diagnosis

4. The clinical features of histoplasmosis are practically same as that of Tuberculosis
5. There is need to screen both ART-experienced and ART-naïve HIV-infected patients with Advanced HIV disease with suggestive features for histoplasmosis
6. Persons at risk include those living around orchards
7. Persons at risk include construction site workers
8. Infections are commonly transmitted from person to person
9. Clinical features can be used to make a diagnosis of disseminated histoplasmosis in AHD patients
10. All HIV-infected patients regardless of CD4+ cell count level should be screened
11. Representative sample for laboratory diagnosis includes urine
12. Histoplasmosis can be diagnosed in the laboratory in less than 10 mins

Treatment

13. In ART- naïve patients that test positive for histoplasmosis, withhold initiation of ART for two weeks while treating patients with antifungals.
14. In severe cases of disseminated histoplasmosis, treat with liposomal amphotericin B
15. In mild to moderate cases of disseminated histoplasmosis treat with itraconazole
16. In mild to moderate cases of disseminated histoplasmosis treat with fluconazole
17. Steroids are contraindicated in patients being treated for histoplasmosis
18. There is no challenge of drug–drug interaction of antifungals and ARTs
19. Stop antifungals once symptoms disappear
20. Stop antifungals once the CD4 cell count is above 200 cells/cm
