# Supplementary material for: Serious Fungal Diseases: Assessing the Impact of Training Clinicians and Laboratory Scientists in Nigeria
Source: Open Forum Infect Dis. 2026 Jul 29;13(8):ofag442. doi: 10.1093/ofid/ofag442 (PMC13439316; doi:10.1093/ofid/ofag442)
Supplement: Supplementary file 1 [file ofag442_supplementary_data.zip › Supplementary_file_Members_of_NIFUSCAB.pdf]

MEMBERS OF THE NIGERIA FUNGAL DISEASES SURVEILLANCE AND CAPACITY BUILDING (NIFUSCAB) PROGRAM COMMITTEE

Surnames in block letters. Titles written as a prefix.

NAME	POSITION	INSTITUTION
Prof. Rita OLADELE	Principal Investigator	College of Medicine, University of Lagos, Lagos State
Prof. Folasade OGUNSOLA	Co-principal Investigator	College of Medicine, University of Lagos, Lagos State
Dr. Damilola AKINLAWON	Program Manager	Lagos University Teaching Hospital, Lagos State
Dr. Olufunmilola MAKANJUOLA	Site Investigator	University Teaching Hospital, Ibadan, Oyo State
Dr. Ubong UDOH	Site Investigator	University of Calabar Teaching Hospital, Cross River State
Dr. Iriagbonse OSAIGBOVO	Site Investigator	University of Benin Teaching Hospital, Edo State
Dr. Philip NWAJIOBI-PRINCEWILL	Site Investigator	National Hospital Abuja
Prof. Yahaya MOHAMMED	Site Investigator	Usmanu Danfodiyo University Teaching Hospital, Sokoto State
Dr. Ifeyinwa NWAFIA	Site Investigator	University of Nigeria Teaching Hospital, Enugu State
Prof. Abayomi FADEYI	Site Investigator	University of Ilorin Teaching Hospital, Ilorin, Kwara State
Dr. Usman UMAR	Site Investigator	Aminu Kano Teaching Hospital, Kano State
Dr. Jonah PETER	Site Investigator	University of Abuja Teaching Hospital, Gwagwalada, Abuja
Mrs. Folakke PETERS	Lead Laboratory scientist	Lagos University Teaching Hospital, Lagos State
